# Cannulation strategies for percutaneous extracorporeal membrane oxygenation in adults

**DOI:** 10.1007/s00392-015-0941-1

**Published:** 2015-11-25

**Authors:** L. Christian Napp, Christian Kühn, Marius M. Hoeper, Jens Vogel-Claussen, Axel Haverich, Andreas Schäfer, Johann Bauersachs

**Affiliations:** Cardiac Arrest Center, Department of Cardiology and Angiology, Hannover Medical School, Carl-Neuberg-Str. 1, 30625 Hannover, Germany; Department of Cardiothoracic, Transplantation and Vascular Surgery, Hannover Medical School, Hannover, Germany; Department of Respiratory Medicine and German Center of Lung Research (DZL), Hannover Medical School, Hannover, Germany; Institute for Diagnostic and Interventional Radiology, Hannover Medical School, Hannover, Germany

**Keywords:** Cardiogenic shock, Heart failure, ECMO, Extracorporeal circulation, Mechanical circulatory support

## Abstract

Extracorporeal membrane oxygenation (ECMO) has revolutionized treatment of severe isolated or combined failure of lung and heart. Due to remarkable technical development the frequency of use is growing fast, with increasing adoption by interventional cardiologists independent of cardiac surgery. Nevertheless, ECMO support harbors substantial risk such as bleeding, thromboembolic events and infection. Percutaneous ECMO circuits usually comprise cannulation of two large vessels (‘dual’ cannulation), either veno-venous for respiratory and veno-arterial for circulatory support. Recently experienced centers apply more advanced strategies by cannulation of three large vessels (‘triple’ cannulation), resulting in veno-veno-arterial or veno-arterio-venous cannulation. While the former intends to improve drainage and unloading, the latter represents a very potent method to provide circulatory and respiratory support at the same time. As such triple cannulation expands the field of application at the expense of increased complexity of ECMO systems. Here, we review percutaneous dual and triple cannulation strategies for different clinical scenarios of the critically ill. As there is no unifying terminology to date, we propose a nomenclature which uses “A” and all following letters for supplying cannulas and all letters before “A” for draining cannulas. This general and unequivocal code covers both dual and triple ECMO cannulation strategies (VV, VA, VVA, VAV). Notwithstanding the technical evolution, current knowledge of ECMO support is mainly based on observational experience and mostly retrospective studies. Prospective controlled trials are urgently needed to generate evidence on safety and efficacy of ECMO support in different clinical settings.

## Introduction

Extracorporeal assist systems are increasingly used for treatment of severe heart and lung failure. The first published report of successful extracorporeal support dates back to 1972 [1]. Since then many technical improvements of tubings, surfaces, oxygenators and other components contributed to a broad use of extracorporeal support systems worldwide. Recently randomized as well as observational studies have demonstrated no significant benefit of intra-aortic balloon pumps in patients with shock during acute myocardial infarction [2, 3]. Thus the frequency of use of ECMO and other systems will likely increase in the future, underlining the need of systematic studies for every form of mechanical support.

In most cases an ECMO circuit comprises two large-bore cannulae in a veno-venous or veno-arterial configuration. During veno-venous ECMO blood is percutaneously drained via a cannula from the right atrium, oxygenated and decarboxylated in a dedicated extracorporeal rotor/oxygenator device and returned via a second cannula to the right atrium. It supports respiratory function and is classically employed during treatment of severe acute respiratory distress syndrome (ARDS). In contrast, the same extracorporeal unit can also be used for providing circulatory support in severe heart failure. In this case blood is again drawn from the venous system but returned to the patient’s arterial system, which is called veno-arterial cannulation. Here ECMO primarily provides hemodynamic support, while the effect on oxygenation depends on arterial and venous cannulation sites, the patient’s cardiac output and respiratory function. In this veno-arterial ECMO is essentially different from veno-venous ECMO.

Percutaneous cannulation and technical improvements of all parts of the ECMO unit have enabled a very quick setup of the system. Nevertheless, ECMO is an invasive life support system, with substantial risk of adverse events like bleeding, vascular complications, thromboembolic events and infection [4]. As such its use should be restricted to selected patients and experienced teams. In principle, ECMO can be used in a *bridge*-*to*-*recovery* strategy, e.g., to replace lung or heart function while these organs recover. In a different approach ECMO bridges organ function until the failing organ is replaced by transplantation (*bridge*-*to*-*transplantation)* or a permanent assist device (e.g., a surgically implanted left ventricular assist device), also referred to as *bridge*-*to*-*destination*. Another strategy is *bridge*-*to*-*decision*, when initial hemodynamic stabilization by the ECMO circuit is necessary to allow for delayed reevaluation and definition of the therapeutic goal.

In addition to dual cannulation, experienced centers have introduced triple cannulation under special circumstances. This concept expands the field of use, but also increases the complexity of an ECMO system. Unfortunately there is no common nomenclature applicable to triple cannulation yet. In every case it is important to consider that ECMO, especially a circuit with arterial cannulation, requires a multidisciplinary and experienced team to limit the potential hazards of initiation, maintenance and weaning of ECMO. The Extracorporeal Life Support Organization (ELSO) has published guidelines on indications, use and weaning from ECMO support in children and adults [5]. Large prospective clinical trials investigating efficacy of ECMO are sparse, even if several smaller studies and case series suggest efficacy and reasonable safety. This may in part be explained by the lifesaving effect of ECMO and the related difficulties to build a control group.

In the present review we summarize current indications, pathophysiology and strategies for percutaneous single, dual and triple cannulation ECMO support and propose a unifying and unequivocal nomenclature for ECMO cannulation. It has to be noted that other extracorporeal systems apart from and technically different to ECMO are available; however, these are not the focus of the present review and are described elsewhere [6, 7].

## Dual cannulation

Dual cannulation ECMO comprises veno-venous and veno-arterial ECMO, which have profound differences in the setup and the consequences for support and monitoring. The description of triple cannulation, which requires understanding of dual cannulation, will follow thereafter.

### Veno-venous cannulation

**Fig. 1:**
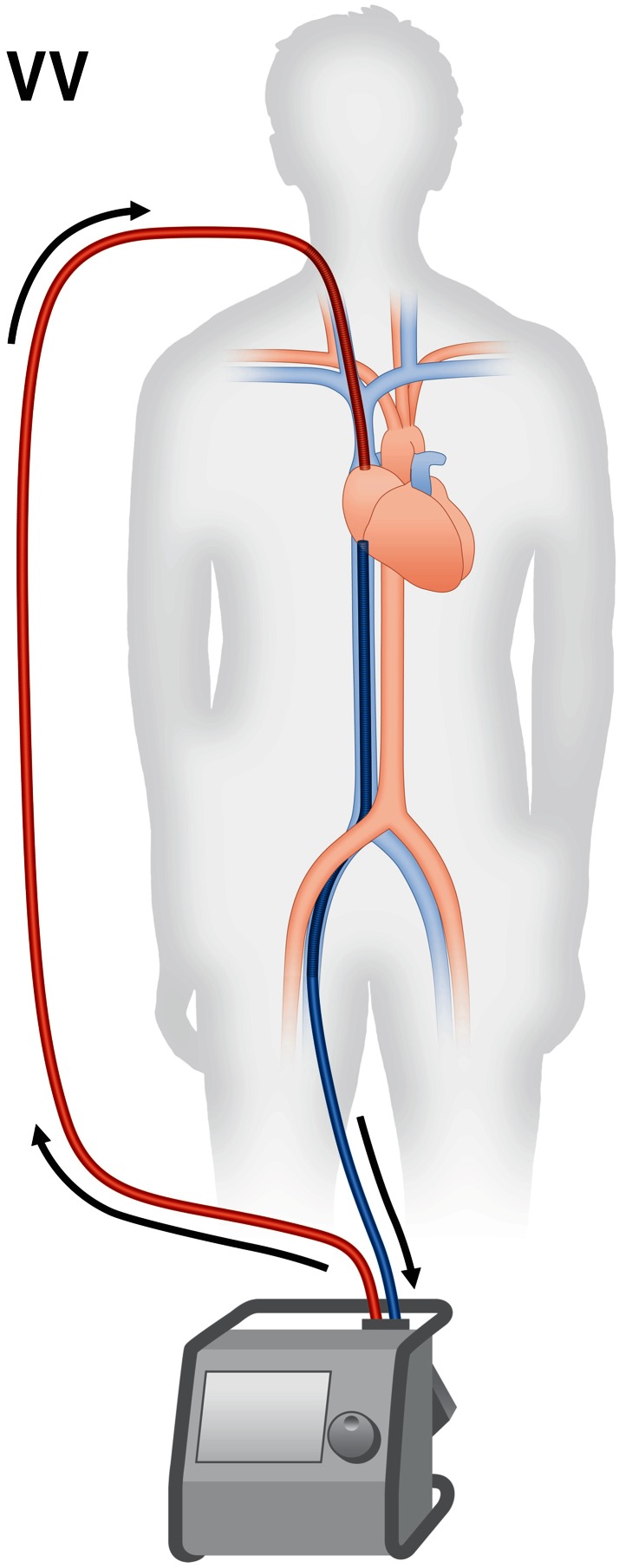
Veno-venous ECMO (VV). Blood is drained from the right atrium and the inferior vena cava, oxygenated and decarboxylated in an extracorporeal rotor/oxygenator device and returned to the right atrium

During veno-venous ECMO deoxygenated blood is drained from a large vein, oxygenated and decarboxylated in an extracorporeal device and returned to the right atrium (Fig. 1). By this, preoxygenated blood enters the pulmonary circuit and provides systemic oxygenation.

#### Indication and clinical studies

The common indication for veno-venous ECMO is ARDS [8], with the intention to provide extracorporeal gas exchange while a protective ventilation strategy allows for lung rest and recovery. Usually ECMO is considered in patients with severe forms of ARDS, and the ELSO recommends ECMO initiation with a Horovitz index below 80. However, many centers start at earlier timepoints, such as a Horovitz index below 100–150 or uncompensated acidosis (pH < 7.2). However, optimal timing, duration and weaning of ECMO have not been investigated in large prospective trials yet. Early trials could not demonstrate a survival benefit of ECMO in ARDS patients [9, 10]. These trials have been a matter of intense debate for different aspects, such as the fact that ventilator settings were not adapted after ECMO initiation, i.e., lung protective ventilation was not performed. In contrast, the conventional ventilatory support versus extracorporeal membrane oxygenation for severe adult respiratory failure (CESAR) trial demonstrated safety and efficacy of veno-venous ECMO compared to conventional ventilation in ARDS patients [11], albeit the trial design has been discussed controversially [12]. Nevertheless, veno-venous ECMO has gained a central role in ARDS with a low Horovitz index, and the emergence of H1N1 has further strengthened the role of ECMO as a lifesaving tool in severe lung failure [13]. Recently the use of veno-venous ECMO in non-intubated patients (“awake-ECMO”) has gained attention, mostly in patients with terminal lung disease awaiting transplantation in a *bridge*-*to*-*transplant* strategy [14] or with ARDS in a *bridge*-*to*-*recovery* strategy [15].

A relative contraindication for veno-venous ECMO are bleeding disorders, since all ECMO configurations require systemic anticoagulation [5]. The use of veno-venous ECMO is contraindicated in patients with terminal respiratory failure, once there is no perspective of organ recovery or lung transplantation.

#### Technical aspects

For veno-venous ECMO usually the femoral and jugular veins are used as vascular access, with the former for drainage and the latter for supply (Fig. 1). Sufficient diameters of right-sided femoral and jugular veins usually allow introduction of ECMO cannulas without problems due to the straight route. The correct position of both cannula tips is the border between the right atrium and the superior and inferior caval veins, respectively (Fig. 1). Malposition may facilitate recirculation, i.e., drainage of freshly oxygenated blood back into the extracorporeal circuit, which may become a substantial problem during therapy. Hence it is essential to verify optimal cannula position by fluoroscopy, chest X-ray or transesophageal echocardiography [16], and modifying the tip of the supplying cannula [17] or positioning it in the right ventricle [18] have been proposed to reduce recirculation.

#### Pathophysiology

In principle, veno-venous ECMO drains blood from a caval vein and returns an equal volume of oxygenated and decarboxylated blood by the other caval vein back to the right atrium. Hence oxygen saturation in the central aorta is the result of a mixture of ECMO-derived blood and residual venous blood returning to the pulmonary circulation, and remaining gas exchange in the lungs. The contribution of both the ECMO and the lung to the final aortic oxygen content varies from patient to patient and over time. Eventually all organs are perfused with approximately the same oxygen saturation, thus a radial or femoral arterial line on either side of the body are sufficient for assessing systemic oxygenation. This is in sharp contrast to all configurations with an arterial cannulation (see below). Another striking difference is that veno-venous ECMO, while profoundly supporting gas exchange, does not influence hemodynamics: The volume of blood drained from the right atrium is replaced by an equal volume of blood from the supplying cannula, resulting in a neutral volume and pressure balance in the right atrium (Table 1). Notwithstanding, during veno-venous ECMO function of the right heart must be closely monitored, and right heart failure in patients with veno-venous ECMO is a potential indication for veno-arterio-venous ECMO (triple cannulation, see below).

**Table 1:** Hemodynamic changes during ECMO support depends on the cannulation mode

Strategy	Right atrial pressure	Left ventricular end-diastolic pressure^a^	Systemic blood pressure	LV afterload	Catecholamine dosing	
					Vasopressors	Inotropes
Veno-venous	↔	↔	↔	↔	↔–↓^b^	↔
Veno-arterial	↓–↓↓	Varies (should decrease)	↑↑	↑↑	↓	↓
Veno-veno-arterial	↓↓	Varies (should decrease)	↑↑	↑↑	↓	↓
Veno-arterio-venous	Varies	↑	↑	↑	Varies	Varies

While VV-ECMO is largely neutral in this context, all cannulations with arterial access profoundly influence venous and arterial pressures by modified flow. Much of the information in this table is based on experience and requires formal confirmation by dedicated studies

^a^Effects vary upon function of the aortic valve

^b^May decrease with improvement of metabolic status by enhanced gas exchange

#### Upper body veno-venous cannulation

**Fig. 2:**
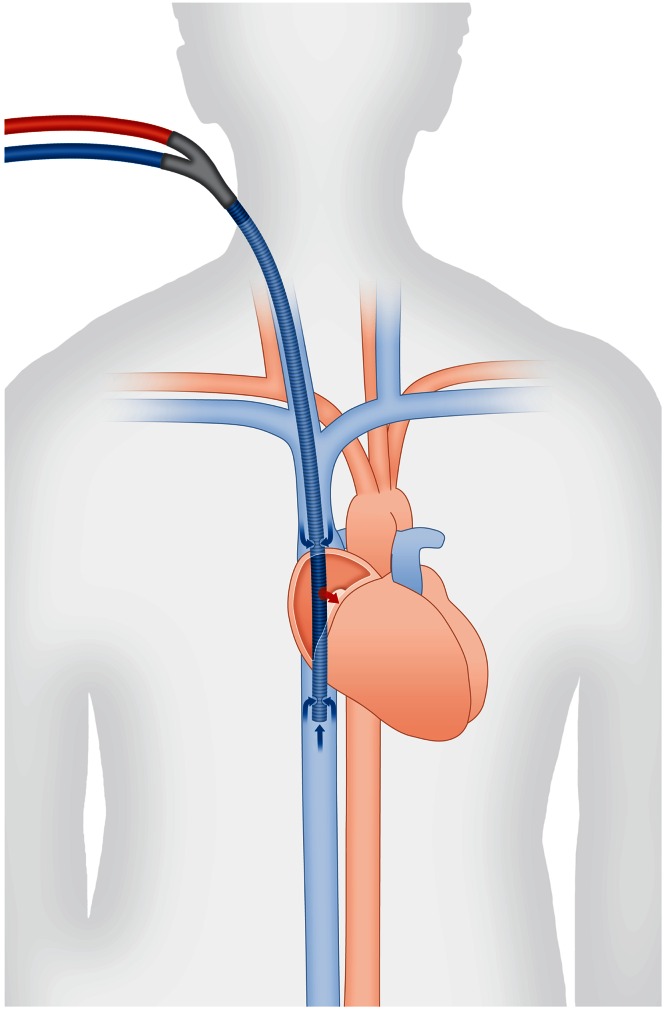
Bicaval dual-lumen cannula. This cannula allows for parallel drainage and supply through one tubing with two lumina during veno-venous ECMO. It thus requires only one large access vein and minimizes recirculation by directed supply towards the tricuspid valve (*red arrow*), spatially separated from the inflow (*blue arrows*)

A promising recent development is to cannulate only veins of the upper body, in particular by using a bicaval dual-lumen cannula (Fig. 2) [19]. This special cannula drains blood from the superior and inferior caval veins, and supplies oxygenated blood to the mid-right atrium directed to the tricuspid valve, thereby minimizing recirculation. A dual-lumen cannula requires puncture of only one large vessel, which is a great advantage in terms of bleeding risk. Upper body cannulation potentially allows for discontinuation of mechanical ventilation and awake-ECMO, active physical therapy and mobilization on the intensive care unit. Despite these benefits there is a risk of right atrial or ventricular perforation of the cannula, which can be reduced by fluoroscopy or echocardiography guided placement, but needs to be determined in larger studies [20]. Furthermore, currently available dual-lumen cannulas are limited in terms of maximal blood flow and hemolysis may emerge on higher flow rates.

### Veno-arterial cannulation

**Fig. 3:**
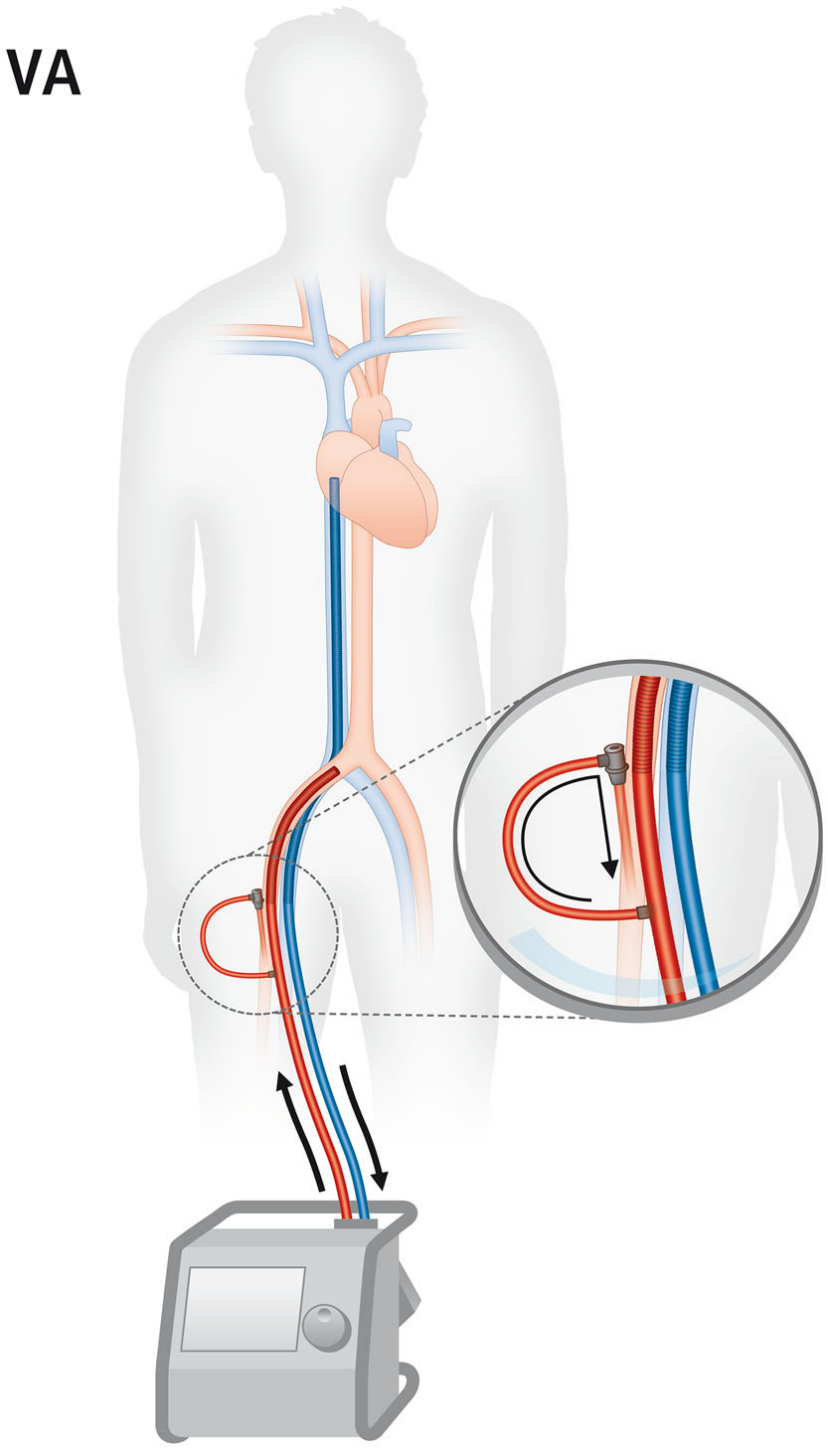
Veno-arterial ECMO (VA). Blood is drained from the right atrium, oxygenated and decarboxylated in the ECMO device and returned to the iliac artery towards the aorta. Note the modified position of the venous cannula tip compared to veno-venous ECMO. Cannulation of the femoral artery requires an additional sheath for perfusion of the leg downstream of the cannulation site (*inset*)

The second important field of use for ECMO is circulatory support in severe acute or decompensated chronic heart failure. Current guidelines recommend early evaluation for mechanical support in patients with cardiogenic shock [21]. For circulatory support veno-arterial cannulation is performed, which differs from veno-venous cannulation in that reoxygenated and decarboxylated blood is returned not to the right atrium but to a large artery towards the aorta (Fig. 3). This extracorporeal right-to-left-shunt unloads the failing heart by preload reduction and adds a stable blood flow of 3–7 l/min to the arterial system, with the intention to maintain a critical blood pressure for end organ perfusion (Table 1). In this context, it is a common misapprehension that ECMO provides pressure support. In contrast, increased blood pressure during veno-arterial ECMO is only a result of increased flow, is as such secondary and depends on vascular resistance and filling. Accordingly, vasopressors and volume therapy have to be carefully adjusted during veno-arterial ECMO.

#### Indication and clinical studies

Veno-arterial ECMO can be used in a variety of conditions. The most frequent indications are failure to wean from cardiopulmonary bypass or early decompensation after cardiac surgery, referred to as postcardiotomy cardiogenic shock [22]. The classical non-surgical indication is cardiogenic shock caused by myocardial infarction [23], decompensated non-ischemic heart-failure [24] or fulminant myocarditis [25], in many cases in a *bridge*-*to*-*recovery* strategy. It is further employed during pulmonary embolism prior to embolectomy [26, 27], or in a *bridge*-*to*-*transplantation* strategy for right ventricular failure during decompensated pulmonary arterial hypertension [28]. Another indication is stabilization of patients with cardiogenic shock to enable their transport to a tertiary cardiovascular center [29]. For this application transportable ECMO systems are available. Veno-arterial ECMO has also been successfully used in a provisional setting for high-risk percutaneous coronary intervention [30]. However, in elective high-risk coronary interventions a percutaneous microaxial pump appears to be equally effective with lower procedural risk [31].

Veno-arterial ECMO can be useful for preconditioning the patient prior to implantation of a permanent left ventricular assist device (LVAD) [32], or in a *bridge*-*to*-*transplantation* setting. It can be continued until patients are awake, e.g., to evaluate neurological outcome after resuscitation (*bridge*-*to*-*decision*), or even be inserted in completely awake patients [28]. After lung transplantation for pulmonary hypertension, veno-arterial ECMO is sufficient for bridging the early postoperative phase while the heart is not ready to manage reconstituted left ventricular preload [33]. Recently the use during resuscitation [34] is increasing, with impressive outcome data: in an observational study of 117 patients without spontaneous ROSC after prolonged resuscitation in whom ECMO was initiated, 15 % survived with favorable neurological outcome [35]. However, extracorporeal cardiopulmonary resuscitation should be considered primarily in scenarios with a reasonable exit strategy, e.g., embolectomy in pulmonary embolism or emergency coronary revascularization [36].

Overall, despite promising data for veno-arterial ECMO from smaller studies, large prospective studies are not reported. Contraindications for arterial cannulation can arise from aortic dissection, aortic regurgitation, left ventricular thrombi or bleeding disorders.

#### Technical aspects

For veno-arterial ECMO usually a femoral vein and the ipsilateral femoral artery are used for vascular access (Fig. 3). The venous cannula may also be placed into a jugular vein. The correct position of the venous cannula tip is the mid-right atrium (Fig. 3) to enable homogenous drainage of venous blood from both caval veins. If placed in the femoral artery, the arterial cannula should be fully introduced resulting in a tip position in the common iliac artery in adults. Upper body cannulation is also possible (see below).

#### Pathophysiology

Once the femoral artery is cannulated during ECMO, some essential differences to veno-venous ECMO have to be considered.

**Fig. 4:**
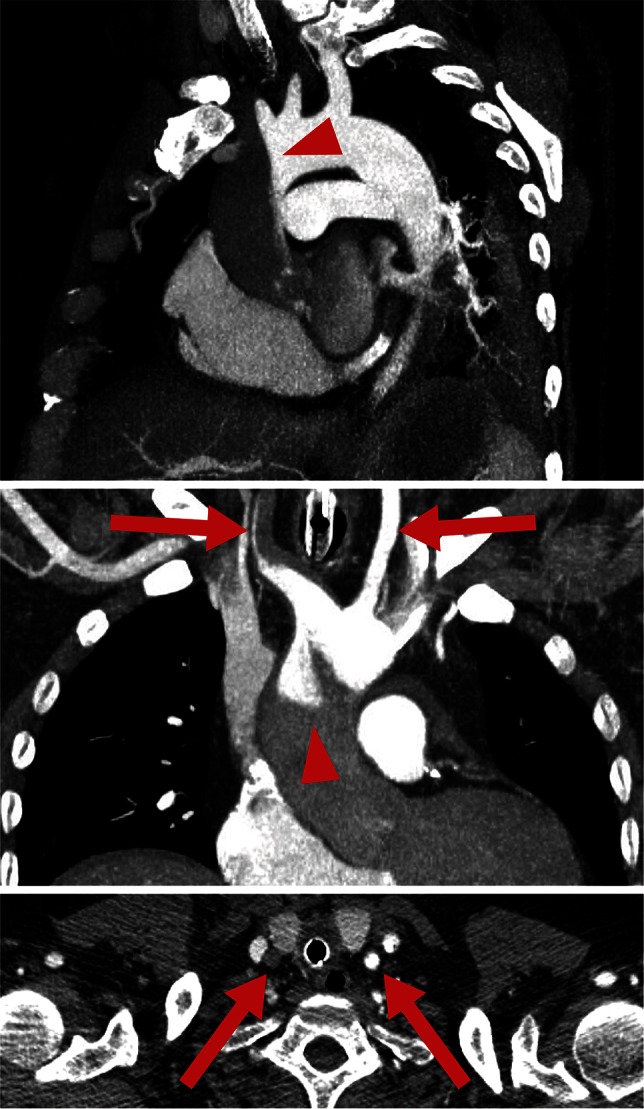
Watershed phenomenon during veno-arterial ECMO visualized by computed tomography. Antegrade blood flow (low contrast) from the heart competes with retrograde blood flow (high contrast) from the ECMO in the aorta, resulting in a watershed phenomenon (*arrowhead*). Here computed tomography of a patient with pulmonary embolism and reduced cardiac output demonstrates a rather proximal watershed, leading to perfusion of the right carotid artery with “heart blood” (*dark*) and the left carotid artery with “ECMO blood” (*bright*, *arrows*). *Upper panel* sagittal oblique maximum intensity projection (MIP), *middle panel* coronal oblique MIP, *lower panel* transverse plane

The first and most important aspect is the so-called watershed phenomenon (Fig. 4): if the failing heart is not able to ensure a critical blood pressure for organ perfusion, flow support by the ECMO unit will result in enhanced blood pressure as long as the vascular system has sufficient resistance (Table 1). However, in most patients on a veno-arterial ECMO the left ventricle still has some output and thus delivers an antegrade blood flow towards the descending aorta. This ‘native’ flow meets the retrograde blood flow from the arterial ECMO cannula at a point called the ‘watershed’ [26]. It is located somewhere between the ascending aorta and the renal arteries in most cases. Importantly, the particular location of the watershed is determined by the competition between left ventricular output and ECMO flow and thus varies during therapy [37] and between patients. In the presence of an antegrade flow through the aortic valve the coronaries and mostly the first branches from the aortic arch will be perfused with blood originating from the left ventricle. All areas distal to the watershed, i.e., the lower half of the body including the kidneys, receive blood oxygenated by the ECMO unit. While oxygen saturation of ECMO-derived blood will be nearly always sufficient, oxygen saturation of blood originating from the left ventricle depends on respiratory function of the lung, which can be severely compromised by edema, pneumonia or other pulmonary conditions. Accordingly, respiratory failure during veno-arterial ECMO may result in hypoxic damage of the heart and brain-despite good perfusion pressure. Therefore, it is mandatory to establish an arterial line at the right upper half of the body (preferably at the right radial artery) for monitoring upper body oxygenation. A femoral arterial line would reflect ECMO oxygenation and is therefore never sufficient for monitoring brain oxygenation. Rarely, the watershed may be located more proximally in the ascending aorta just between the coronaries and the right brachiocephalic trunk [26], which renders monitoring of oxygen saturation for the coronaries virtually impossible. This condition is a possible indication for veno-arterio-venous cannulation (triple cannulation, see below) to ensure coronary oxygenation or implantation of a microaxial pump (see below) to enhance antegrade flow. For the above described reasons it is essential to evaluate pulse pressure (as a surrogate of left ventricular output) and upper body oxygenation immediately after veno-arterial ECMO insertion and continuously thereafter [37].

Second, femoral arterial cannulation reduces perfusion distal to the puncture site. This potentially causes lower limb ischemia and may result in vascular surgery, compartment decompression or amputation [4, 38]. Therefore, an additional sheath is required to ensure distal arterial perfusion (Fig. 3, inset). This sheath should ideally be placed before introducing the arterial cannula, as arterial filling distal to the cannula may be diminished after cannulation. If ECMO cannulation is performed in the cath lab, we prefer to place the antegrade sheath with angiographic guidance. Of note, arterial cannulation generally harbors the risk of arterial injury, e.g., by dissection, rupture or occlusion [39], potentially requiring emergency vascular surgery.

Third, left ventricular distension and pulmonary congestion may occur after in patients with veno-arterial ECMO, especially in cases of extremely low left ventricular output or aortic regurgitation. Retrograde aortic ECMO flow increases afterload, and some blood still arrives in the left heart returning from bronchial and thebesian veins [40] even with full ECMO speed, ultimately resulting in left ventricular distension. In such cases a second venous draining cannula for enhanced preload reduction can be helpful (veno-veno-arterial cannulation, see below) [5]. A novel promising solution to compensate for insufficient or missing antegrade flow across the aortic valve is percutaneous left ventricular unloading by a microaxial pump (Impella^**®**^) in addition to veno-arterial ECMO [41]. Such a pump can easily be implanted in the catheterization laboratory without the need for open surgery, facilitating profound left ventricular support and decompression.

It has to be noted that the above described aspects are of critical importance during ECMO with femoral arterial cannulation; however, they do not apply to central and only in part to subclavian arterial cannulation.

#### Upper body veno-arterial cannulation

Some centers have described accessing the right internal jugular vein and the subclavian artery for veno-arterial ECMO [42]. This results in upper body cannulation, allowing for awake-ECMO, mobilization and active physical therapy. In this case the watershed problem is reduced, at least with respect to brain oxygenation. However, subclavian artery cannulation requires direct surgical arterial access by applying a vascular end-to-side graft, is much more invasive and harbors the risk of injuring vessels or nerves of the arm.

## Triple cannulation

Triple cannulation is a novel and special form of ECMO support, which is usually employed as an “upgrade” of an existing veno-venous or veno-arterial ECMO circuit. Triple cannulation may either be instituted as veno-veno-arterial or veno-arterio-venous cannulation, which are essentially different in terms of circulatory and respiratory support as well as associated ventilator and medical management. While more and more centers recently apply triple cannulation in selected patients, only few publications exist in the literature, which are summarized in Table 2.

**Table 2:** Publications on triple cannulation ECMO support

Strategy	Patients with triple cannulation	Characteristics	Outcomes
*Veno-veno-arterial* (*Fig.* 5)			
Ford and Atkinson [45]	*n* = 1	A 3000-g 37-week gestation child was born by vaginal delivery and developed respiratory failure from congenital diaphragmal hernia. Veno-arterial ECMO was initiated, but within 24-h hemodynamic support was insufficient due to limited flow through the venous cannula (low bladder pressure, low blood pressure, low central venous oxygenation of 60 %). A third cannula was inserted into the right common iliac vein by cutdown. After veno-veno-arterial ECMO had started central venous saturation increased up to 79 %. Total ECMO support lasted 5 days	The patient underwent surgery for diaphragmal hernia, could be weaned from ECMO and the ventilator and could be discharged home after 31 days in hospital
Hou et al. [44]	Sheep model	Animal study on the effects of different drainage locations during ECMO support. While veno-arterial ECMO with inferior vena cava drainage was running, acute respiratory failure was initiated. This led to severe upper body hypoxemia, with no significant effect on blood pressure. Repositioning the venous drainage cannula to the superior vena cava strongly increased aortic oxygen saturation from 35 to 75 % and thereby reverted upper body hypoxemia	Drainage from the superior vena cava strongly improved systemic oxygen saturation, strongly suggesting that bicaval drainage is sufficient to disrupt the “two-circulation-syndrome”
ELSO [5]	Guideline	Guideline for ECMO support in adults of the Extracorporeal Life Support Organization (ELSO). The guideline mentions the option to add a cannula from the superior vena cava for improved venous drainage	
*Veno-arterio-venous* (*Fig.* 6)			
Madershahian et al. [51]	*n* = 1	Three patients with veno-arterial ECMO due to ARDS after polytrauma. One of them had persistent upper body hypoxemia and needed conversion to veno-arterio-venous ECMO, which led to an increase of pH from 7.2 to 7.45, lung compliance from 15 to 40 ml/mbar and oxygen saturation from 70 to 95 %. Total ECMO support lasted 4.7 ± 1.1 days	No ECMO-related complications were reported. All patients were successfully weaned from ECMO and later on from ventilation and could be discharged
Stöhr et al. [53]	*n* = 11	30 patients with ARDS from pneumonia (*n* = 8), lung graft failure (*n* = 4) or primary lung disease (*n* = 5), trauma (*n* = 2), post-surgery (*n* = 7), sepsis (*n* = 2) or near-drowning (*n* = 1). Initially 18 had veno-venous, nine had veno-arterial and three had veno-arterio-venous cannulation. Subsequently, eight were upgraded from veno - venous or veno-arterial to veno-arterio-venous ECMO, two were set from veno-venous to veno-arterial ECMO. 11 patients had subclavian arterial cannulation. Hemodynamic measures over time are not provided. Mean duration of ECMO support was 7.5 ± 7.2 days	Bleeding occured in eight patients (one venous and seven arterial) and hyperperfusion and leg ischemia and wound healing complications in one patient each. 15 patients died during ECMO support, one died after ECMO explantation. Mortality was higher -in the veno-venous cohort (63 %) and the veno-arterial cohort (75 %) than in the veno-arterio-venous cohort (27 %). Overall 30-day mortality rate was 53 %. One patient was bridged to lung transplantation. During a mean follow-up of 21 months three patients died
Kustermann et al. [46]	*n* = 1	30-year-old patient with community-acquired pneumonia who developed ARDS and severe septic cardiomyopathy. Veno-arterial ECMO was initiated, but was expanded to veno-arterio-venous cannulation because of a remaining low Horovitz index of 130 on ECMO support. FiO_2_ and ventilation pressures could be reduced and 1 day later ECMO was downgraded to veno-venous in the presence of improvement of left ventricular function (LVEF from 10 to 45 %). Total ECMO support lasted for 7 days	No ECMO-related complications were reported. Successful weaning off ECMO was followed by transfer to the referring hospital and complete weaning from ventilation
Moravec et al. [48]	*n* = 3	74-year-old patient with pulmonary hypertension related to pulmonary fibrosis, who developed pneumonia, sepsis and subsequent shock. Initial veno-arterial ECMO was expanded to veno-arterio-venous ECMO with a jugular Shaldon catheter for ARDS. FiO_2_ decreased from 100 to 45 %, with a nearly doubled PaO_2_. Total ECMO support lasted 9 days. 59-year-old obese patient with cardiogenic shock, refractory to medical therapy, who was resuscitated during cardiac catheterization and received an IABP. He was stabilized with veno-arterial ECMO, but developed ARDS and a jugular Shaldon catheter as third cannula was implanted for venous preoxygenation. FiO_2_ decreased from 100 to 40 %, with a more than doubled PaO_2_. Total ECMO support lasted 13 days. A third patient was reported, who received veno-arterio-venous ECMO with standard ECMO cannulae instead of a Shaldon catheter. In this patient ECMO was withdrawn after 12 days and the patient was discharged from hospital later	No ECMO-related complications were reported. All three patients could successfully be weaned from ECMO support. The first patient died later on from lung fibrosis without the prospect of receiving transplantation, but the second one survived without neurological deficit. The third patient was discharged after weaning from ECMO
Chung et al. [40]	Review	Excellent review emphasizing the various aspects of monitoring during ECMO support. The authors describe the principle of veno-arterio-venous triple cannulation	
Choi et al. [43]	*n* = 1	39-year-old patient with acute myocardial infarction. Veno-arterial ECMO was inserted during cardiopulmonary resuscitation. 5 days after onset of ECMO secondary respiratory failure and subsequent brain hypoxia (upper body hypoxemia) developed. A third cannula was added for preoxygenating venous blood. PaO_2_ increased from 39 to 103 mmHg, SO_2_ from 69 to 89 %. Hemodynamics were not provided in the publication. Duration of ECMO support was 10 days, with 5 days of veno-arterio-venous cannulation	The patient was successfully weaned from ECMO and ventilator and was sent to rehabilitation, with an uneventful recovery at 13-month follow-up
Kim et al. [50]	*n* = 1	Nine patients with ECMO after resuscitation for near-drowning. Seven patients received veno-arterial cannulation, one was converted to veno-venous ECMO in the presence of very good hemodynamics and continued ARDS, and one patient initially received veno-arterio-venous ECMO in the presence of severe ARDS and concomitant cardiac dysfunction. Measures for this single patient are not provided. Mean duration of ECMO support was 7.8 days	All patients were weaned from ECMO, and there were no ECMO-related complications reported. Seven patients survived with a favorable neurological outcome, two patients had irreversible hypoxic brain damage and eventually died
Biscotti et al. [52]	*n* = 21	21 patients with veno-arterio-venous ECMO. 11 patients were set at triple cannulation from the beginning for severe combined cardiorespiratory failure, such as pulmonary embolism, terminal lung disease with cardiac failure, ARDS with cardiogenic shock or LVAD failure. Eight patients had veno-venous ECMO, e.g., for ARDS or cystic fibrosis and were switched to veno-arterio-venous cannulation due to new onset of heart failure. One patient had lung transplantation on veno-arterial ECMO and thereafter received veno-arterio-venous ECMO as a bridge to veno-venous ECMO. One patient had ARDS and experienced upper body hypoxemia during veno-arterial ECMO, which was subsequently expanded to veno-arterio-venous ECMO. Mean duration of ECMO support was 6.5 ± 5.5 days	Seven patients had bleeding. Other complications were oxygenator failure (*n* = 3) or clotting (*n* = 4), cannula thromboses or repositioning. Eight patients died during ECMO, four were weaned from ECMO but died before discharge, nine survived to discharge. Four of 11 who initially had veno-arterio-venous ECMO survived, four of eight converted from veno-venous ECMO survived; and one of two converted from veno-arterial ECMO survived
Ius et al. [47]	*n* = 10	Nine patients with veno-venous ECMO, one patient with veno-arterial ECMO. ECMO was started for ARDS or other forms of respiratory failure. All patients were switched to veno-arterio-venous cannulation for new onset heart failure (right heart failure, pericardial tamponade or mitral regurgitation). Time-to-switch was 2 ± 2.5 days, with a total ECMO support time of 10 ± 4 days	One patient developed pericardial effusion. Three patients had bleeding, and two patients developed leg ischemia. Three patients were successfully bridged to lung transplantation, of which two survived to hospital discharge. Another four were successfully weaned off ECMO, of which three survived to hospital discharge. Three patients died on ECMO support during hospitalization
ELSO [5]	Guideline	Guideline for ECMO support in adults of the Extracorporeal Life Support Organization (ELSO). The guideline offers to convert veno-arterial to veno-arterio-venous cannulation when severe respiratory failure occurs	

ARDS denotes acute respiratory distress syndrome

*FiO*_*2*_ distress syndrome, inspiratory oxygen fraction, *LVEF* left ventricular ejection fraction, *PaO*_*2*_ partial oxygen saturation

### Veno-veno-arterial cannulation

The therapeutic goals of veno-arterial ECMO are circulatory flow support and cardiac unloading by reduction of filling pressures. This can be well monitored by a Swan–Ganz catheter, with pulmonary arterial and capillary wedge pressures as robust markers of filling and unloading. In contrast, vascular resistance calculation and cardiac output measurements by thermodilution will remain unreliable due to right atrial drainage.

**Fig. 5:**
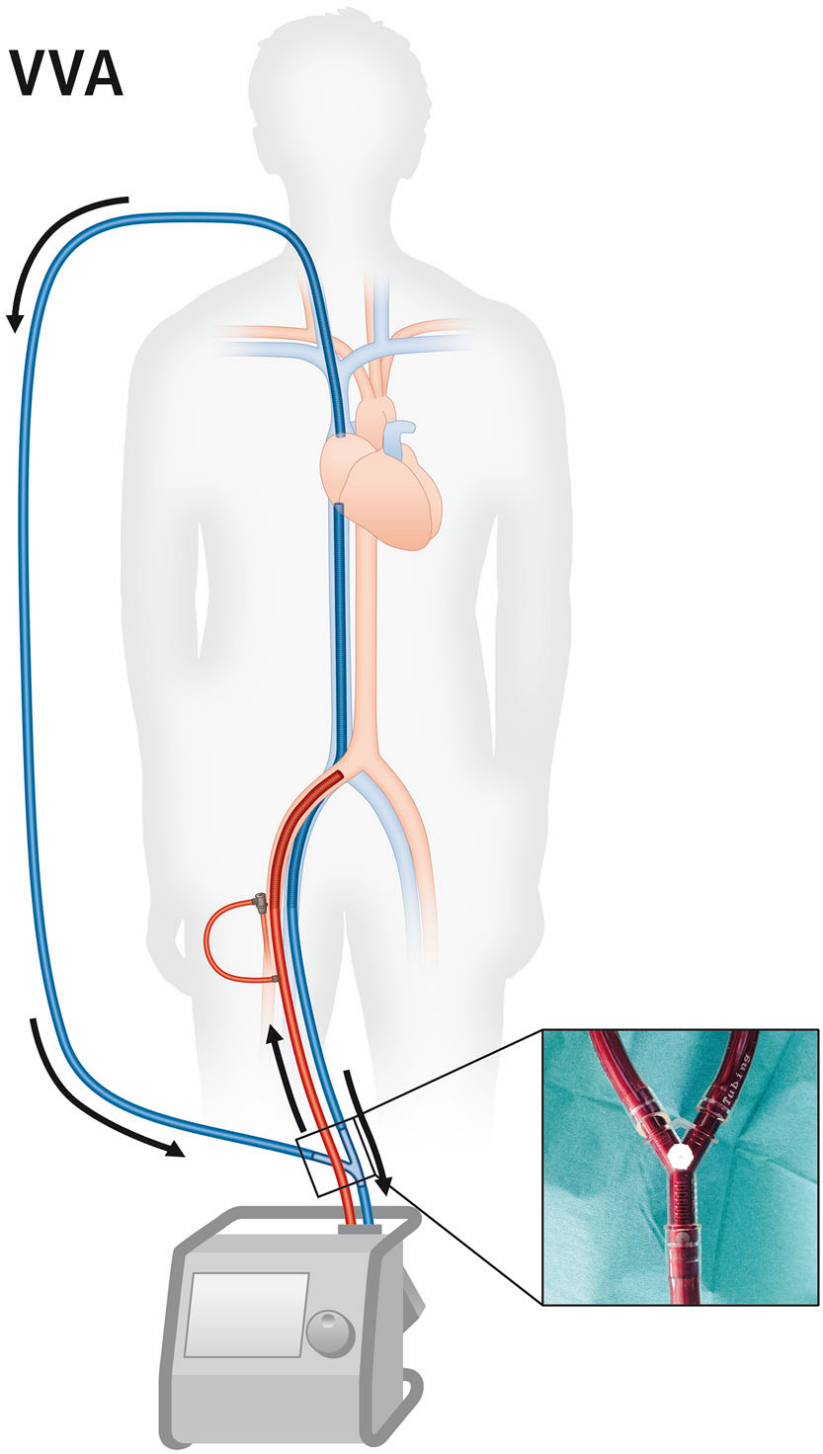
Veno-veno-arterial ECMO (VVA). When unloading by veno-arterial ECMO is not sufficient, a second draining cannula may be necessary. The draining flows from the two venous cannulas are merged outside the body using a Y-connector (*inset*)

In some patients on veno-arterial ECMO respiratory function is not sufficient which potentially results in upper body hypoxemia, also referred to as differential hypoxia, two-circulation syndrome or harlequin syndrome [43, 44]. This phenomenon may further occur in very large patients supported with standard sized cannulae. Then veno-arterial ECMO support can be enhanced by the addition of a second draining cannula, resulting in triple cannulation (two for drainage and one for supply, Fig. 5), which is sufficient to disrupt dual circulation in many cases.

#### Technical aspects and pathophysiology

Unloading by veno-arterial ECMO may be insufficient in selected patients, e.g., in grown-up patients with congenital heart defects and the coexistence of intracardiac shunts and pulmonary arterial hypertension: intracardiac right-to-left shunt and two-circulation syndrome contribute to hypoxemia in the ascending aorta and possibly result in myocardial and cerebral hypoxic damage. Optimized unloading, upper body drainage and shunt reversion may then be achieved by adding a second draining cannula to the system, which drains blood from the right atrium or the right ventricle (veno-veno-arterial ECMO, Fig. 5). Another indication for a second draining cannula may arise from left ventricular distension due to suboptimal drainage during veno-arterial ECMO as described above. Furthermore, in selected cases of veno-arterial ECMO drainage with a single venous cannula may not be sufficient, e.g., in the presence of small vein diameters or hemolysis due to high flows. Then a veno-veno-arterial cannulation strategy can be helpful to increase venous drainage and to enable high flows [40, 45].

The second venous cannula should be placed under echocardiographic guidance. This can best be achieved by fluoroscopy or transesophageal echocardiography. Both venous cannulae are then connected outside the body using a Y-connector (Fig. 5, inset), so that venous blood eventually returns through one tubing to the ECMO unit.

It has to be noted that no study data for veno-veno-arterial ECMO exist and that this configuration has yet been used only in highly selected adult patients and in children (Table 2). Hemodynamic consequences of veno-veno-arterial ECMO are comparable to veno-arterial cannulation (Table 1).

### Veno-arterio-venous cannulation

**Fig. 6:**
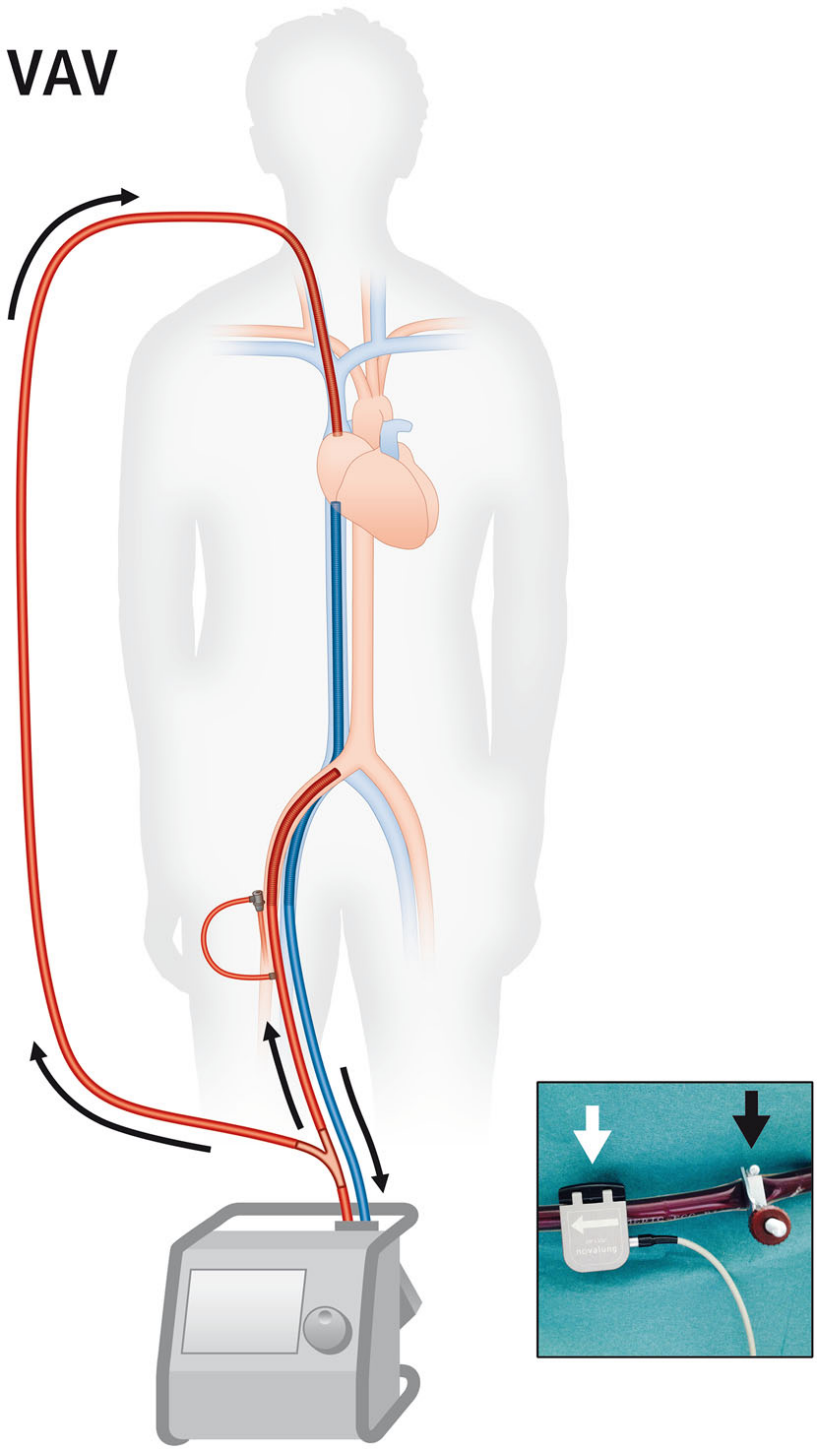
Veno-arterio-venous ECMO (VAV). When circulatory support with veno-arterial ECMO is complicated by respiratory failure or when respiratory support by veno-venous ECMO is complicated by heart failure, a third cannula may be necessary. Both approaches result in one draining and two supplying cannulae. Flow through the supplying cannulae is balanced using an adjustable clamp (*inset*, *black arrow*) and a separate flow sensor (*inset*, *white arrow*)

Recently, veno-arterio-venous configuration has been reported in patients with concomitant lung and heart failure. In this type of cannulation the arterial outflow is divided, with one part towards the aorta and one part towards the right atrium (Fig. 6). In this it combines the advantages and special features of veno-venous and veno-arterial ECMO, providing potent respiratory and circulatory support at the same time. Thus it appears very attractive in selected cases with combined heart and lung failure, such as severe left ventricular failure with secondary pneumonia or right heart decompensation during ARDS.

#### Indication and clinical studies

Veno-arterio-venous ECMO has a potential indication either in cardiac and secondary respiratory failure and vice versa. During heart failure veno-arterial ECMO aims to unload the heart and to maintain arterial blood pressure. However, when respiratory failure develops during ECMO support, e.g., due to pulmonary edema, severe pneumonia or ventilator-associated lung injury, myocardial and cerebral oxygenation may be compromised resulting from the watershed phenomenon described above: The upper body appears cyanotic and the lower body appears pink, also referred to as “two-circulation syndrome” or differential hypoxia [43, 44]. In such situations a third cannula can be added for supplying arterialized blood into the pulmonary circulation.

Another potential indication is heart failure developing in patients on veno-venous ECMO support [46, 47]. In this case, an arterial cannula must be added to provide circulatory support, for maintaining systemic blood pressure and unloading the heart.

To date, a few case series and small observational studies have demonstrated reasonable safety and efficacy of veno-arterio-venous ECMO [43, 46–53] (Table 2); however, prospective or controlled data are not reported yet.

#### Technical aspects

Veno-arterio-venous cannulation is usually implemented as an “upgrade” of a veno-arterial or veno-venous ECMO. In both cases the arterialized ECMO outflow is divided via a Y-connector, for one arterial cannula supplying towards the central aorta and one venous cannula supplying towards the pulmonary circulation (Fig. 6). An adjustable clamp and a separate flow sensor on one of the two outflow tubings allow for balancing the flows between both cannulae (Fig. 6, inset). The preferred position of central venous cannulas in veno-arterio-venous ECMO is the border between the caval veins and the right atrium, like in veno-venous ECMO (Figs. 1, 6). Cannulation can also be achieved with two vascular access points: For this a bicaval dual-lumen cannula is used for venous cannulation. The drainage lumen of the cannula is then connected to ECMO input and the supplying lumen to ECMO output.

#### Pathophysiology

Every patient with a veno-arterio-venous ECMO has an individual demand of arterialized blood flow for each supplying cannula, which will also vary during therapy. It is important to carefully adjust the balance, since every change will influence preload, afterload, the position of the watershed and oxygenation at the same time (Table 1). Therefore, routine control of right and left ventricular filling and systolic function by transthoracic echocardiography is critical during veno-arterio-venous ECMO, especially after modification of the flow balance. As with flow balance, changes in oxygenator and sweep gas settings at the ECMO will influence oxygenation and decarboxylation in both reinfusion cannulas and should thus be carefully adjusted. Respiratory support by this cannulation is usually strong and facilitates lung protective ventilation. However, circulatory support during veno-arterio-venous ECMO is lower (nearly half the support) compared to veno-arterial and veno-veno-arterial cannulation, since not the whole arterial flow is directed towards the aorta [47].

### A unified nomenclature

Triple cannulation has lots of implications for pressure, flow and oxygenation and further increases the complexity of any ECMO unit. So far, to the best of our knowledge, there is no uniform nomenclature for systems with two or three cannulas. In a triple cannulated ECMO one cannula is draining from a vein and one supplying to an artery; however, the third cannula may be a draining or supplying one with completely different consequences. During clinical routine clinicians frequently use the short term “vva-ECMO” or “triple cannulation” irrespective of the nature of the third cannula. This approach is ambiguous and may facilitate dangerous misunderstandings. We thus propose an unequivocal yet short and simple common nomenclature of cannulation strategies (Table 3): we suggest that “A” and all following letters denominate supplying cannulas, since “A” is always a supplying (not draining) arterial cannula. Following this, veno-veno-arterial cannulation would be named “VVA-ECMO”, since it involves two draining venous and one supplying arterial cannulae (Fig. 5). On the other hand, veno-arterio-venous cannulation would be named “VAV-ECMO”, as it comprises one draining venous, one supplying arterial and one supplying venous cannula (Fig. 6). This nomenclature might help to prevent misnomers and misunderstandings during clinical communication.

**Table 3:** A unified nomenclature for ECMO cannulation

Strategy	Figures	Draining cannula^a^	Supplying cannula^a^	Indication
VV	1	Inferior vena cava	Superior vena cava	ARDS
VA	3	Right atrium	Common iliac artery	Postcardiotomy cardiogenic shock Acute decompensated heart failure Cardiogenic shock during AMI or fulminant myocarditis Massive pulmonary embolism with shock High-risk PCI support Extracorporeal resuscitation
VVA	5	Inferior vena cava Superior vena cava (or RV or PA)	Common iliac artery	Insufficient unloading during VA-ECMO Left ventricular distension during VA-ECMO
VAV	6	Inferior vena cava	Common iliac artery Superior vena cava	Respiratory failure during VA-ECMO Cardiogenic shock during VV-ECMO

Letters before “A” are draining cannulas, and “A” and all following letters denominate supplying cannulas. The proposed nomenclature does not consider the arterial sheath for distal leg perfusion and does not change upon use of a bicaval dual-lumen cannula

*AMI* denotes acute myocardial infarction, *ARDS* acute respiratory distress syndrome, *PA* pulmonary artery, *PCI* percutaneous coronary intervention, *RV* right ventricle

^a^Typical place of blood supply/drainage (cannula tip), not place of vascular access/puncture

## Conclusion

Veno-venous (VV) and veno-arterial (VA) ECMO with percutaneous cannulation are increasingly used for mechanical support during severe respiratory and cardiac failure, respectively. Upper body cannulation and awake-ECMO are promising innovative approaches allowing mobilization of the patient. Occasionally experienced centers add a third cannula to an ECMO circuit, either as veno-veno-arterial (VVA) cannulation for improved drainage or as veno-arterio-venous (VAV) cannulation for combining the features of VV- and VA-ECMO. This increases the complexity of the circuit, but gives the opportunity to augment ECMO efficacy in special clinical situations and to rescue patients with severe combined heart and lung failure. We recommend using a unified nomenclature for cannulation as proposed here in order to prevent misunderstandings. Prospective controlled trials are needed to generate robust evidence on safety and efficacy of different ECMO modes in various clinical settings.

## References

[1] Hill JD, O‘Brien TG, Murray JJ, Dontigny L, Bramson ML, Osborn JJ, Gerbode F (1972). Prolonged extracorporeal oxygenation for acute post-traumatic respiratory failure (shock-lung syndrome). Use of the Bramson membrane lung. New Engl J Med.

[2] Thiele H, Zeymer U, Neumann FJ, Ferenc M, Olbrich HG, Hausleiter J, Richardt G, Hennersdorf M, Empen K, Fuernau G, Desch S, Eitel I, Hambrecht R, Fuhrmann J, Bohm M, Ebelt H, Schneider S, Schuler G, Werdan K, Investigators I-SIT (2012). Intraaortic balloon support for myocardial infarction with cardiogenic shock. New Engl J Med.

[3] Zeymer U, Hochadel M, Hauptmann KE, Wiegand K, Schuhmacher B, Brachmann J, Gitt A, Zahn R (2013). Intra-aortic balloon pump in patients with acute myocardial infarction complicated by cardiogenic shock: results of the ALKK-PCI registry. Clin Res Cardiol.

[4] Zangrillo A, Landoni G, Biondi-Zoccai G, Greco M, Greco T, Frati G, Patroniti N, Antonelli M, Pesenti A, Pappalardo F (2013). A meta-analysis of complications and mortality of extracorporeal membrane oxygenation. Crit Care Resusc.

[5] Extracorporeal Life Support Organization: ELSO guidelines. http://www.elsoorg/resources/guidelines

[6] Werdan K, Gielen S, Ebelt H, Hochman JS (2014). Mechanical circulatory support in cardiogenic shock. Eur Heart J.

[7] Ferrari M, Kruzliak P, Spiliopoulos K (2015). An insight into short- and long-term mechanical circulatory support systems. Clin Res Cardiol.

[8] Brodie D, Bacchetta M (2011). Extracorporeal membrane oxygenation for ARDS in adults. New Engl J Med.

[9] Morris AH, Wallace CJ, Menlove RL, Clemmer TP, Orme JF, Weaver LK, Dean NC, Thomas F, East TD, Pace NL, Suchyta MR, Beck E, Bombino M, Sittig DF, Bohm S, Hoffmann B, Becks H, Butler S, Pearl J, Rasmusson B (1994). Randomized clinical trial of pressure-controlled inverse ratio ventilation and extracorporeal CO_2_ removal for adult respiratory distress syndrome. Am J Respir Crit Care Med.

[10] Zapol WM, Snider MT, Hill JD, Fallat RJ, Bartlett RH, Edmunds LH, Morris AH, Peirce EC, Thomas AN, Proctor HJ, Drinker PA, Pratt PC, Bagniewski A, Miller RG (1979). Extracorporeal membrane oxygenation in severe acute respiratory failure. A randomized prospective study. JAMA.

[11] Peek GJ, Mugford M, Tiruvoipati R, Wilson A, Allen E, Thalanany MM, Hibbert CL, Truesdale A, Clemens F, Cooper N, Firmin RK, Elbourne D, Collaboration Ct (2009). Efficacy and economic assessment of conventional ventilatory support versus extracorporeal membrane oxygenation for severe adult respiratory failure (CESAR): a multicentre randomised controlled trial. Lancet.

[12] Sidebotham D (2011). Extracorporeal membrane oxygenation—understanding the evidence: CESAR and beyond. J Extra-corpor Technol.

[13] Pham T, Combes A, Roze H, Chevret S, Mercat A, Roch A, Mourvillier B, Ara-Somohano C, Bastien O, Zogheib E, Clavel M, Constan A, Marie Richard JC, Brun-Buisson C, Brochard L, Network RR (2013). Extracorporeal membrane oxygenation for pandemic influenza A(H1N1)-induced acute respiratory distress syndrome: a cohort study and propensity-matched analysis. Am J Respir Crit Care Med.

[14] Fuehner T, Kuehn C, Hadem J, Wiesner O, Gottlieb J, Tudorache I, Olsson KM, Greer M, Sommer W, Welte T, Haverich A, Hoeper MM, Warnecke G (2012). Extracorporeal membrane oxygenation in awake patients as bridge to lung transplantation. Am J Respir Crit Care Med.

[15] Hoeper MM, Wiesner O, Hadem J, Wahl O, Suhling H, Duesberg C, Sommer W, Warnecke G, Greer M, Boenisch O, Busch M, Kielstein JT, Schneider A, Haverich A, Welte T, Kuhn C (2013). Extracorporeal membrane oxygenation instead of invasive mechanical ventilation in patients with acute respiratory distress syndrome. Intensive Care Med.

[16] Fortenberry JD, Pettignano R, Dykes F, Van Meurs K, Lally DP, Peek G, Zwischenberger JB (2005). Principles and practice of venovenous ECMO. ECMO extracorporeal cardiopulmonary support in critical care.

[17] Bonacchi M, Harmelin G, Peris A, Sani G (2011). A novel strategy to improve systemic oxygenation in venovenous extracorporeal membrane oxygenation: the “chi-configuration”. J Thorac Cardiovasc Surg.

[18] Lindstrom SJ, Mennen MT, Rosenfeldt FL, Salmonsen RF (2012). Veno-right ventricular cannulation reduces recirculation in extracorporeal membrane oxygenation. Perfusion.

[19] Bermudez CA, Rocha RV, Sappington PL, Toyoda Y, Murray HN, Boujoukos AJ (2010). Initial experience with single cannulation for venovenous extracorporeal oxygenation in adults. Ann Thorac Surg.

[20] Chimot L, Marque S, Gros A, Gacouin A, Lavoue S, Camus C, Le Tulzo Y (2013). Avalon(c) bicaval dual-lumen cannula for venovenous extracorporeal membrane oxygenation: survey of cannula use in France. ASAIO J.

[21] McMurray JJ, Adamopoulos S, Anker SD, Auricchio A, Bohm M, Dickstein K, Falk V, Filippatos G, Fonseca C, Gomez-Sanchez MA, Jaarsma T, Kober L, Lip GY, Maggioni AP, Parkhomenko A, Pieske BM, Popescu BA, Ronnevik PK, Rutten FH, Schwitter J, Seferovic P, Stepinska J, Trindade PT, Voors AA, Zannad F, Zeiher A, Guidelines ESCCfP (2012). ESC Guidelines for the diagnosis and treatment of acute and chronic heart failure 2012: The task force for the diagnosis and treatment of acute and chronic heart failure 2012 of the European society of cardiology. Developed in collaboration with the Heart Failure Association (HFA) of the ESC. Eur Heart J.

[22] Maxwell BG, Powers AJ, Sheikh AY, Lee PH, Lobato RL, Wong JK (2014). Resource use trends in extracorporeal membrane oxygenation in adults: an analysis of the nationwide inpatient sample 1998–2009. J Thorac Cardiovasc Surg.

[23] Sheu JJ, Tsai TH, Lee FY, Fang HY, Sun CK, Leu S, Yang CH, Chen SM, Hang CL, Hsieh YK, Chen CJ, Wu CJ, Yip HK (2010). Early extracorporeal membrane oxygenator-assisted primary percutaneous coronary intervention improved 30-day clinical outcomes in patients with ST-segment elevation myocardial infarction complicated with profound cardiogenic shock. Crit Care Med.

[24] Guenther S, Theiss HD, Fischer M, Sattler S, Peterss S, Born F, Pichlmaier M, Massberg S, Hagl C, Khaladj N (2014). Percutaneous extracorporeal life support for patients in therapy refractory cardiogenic shock: initial results of an interdisciplinary team. Interact Cardiovasc Thorac Surg.

[25] Asaumi Y, Yasuda S, Morii I, Kakuchi H, Otsuka Y, Kawamura A, Sasako Y, Nakatani T, Nonogi H, Miyazaki S (2005). Favourable clinical outcome in patients with cardiogenic shock due to fulminant myocarditis supported by percutaneous extracorporeal membrane oxygenation. Eur Heart J.

[26] Hoeper MM, Tudorache I, Kuhn C, Marsch G, Hartung D, Wiesner O, Boenisch O, Haverich A, Hinrichs J (2014). Extracorporeal membrane oxygenation watershed. Circulation.

[27] Belohlavek J, Rohn V, Jansa P, Tosovsky J, Kunstyr J, Semrad M, Horak J, Lips M, Mlejnsky F, Balik M, Klein A, Linhart A, Lindner J (2010). Veno-arterial ECMO in severe acute right ventricular failure with pulmonary obstructive hemodynamic pattern. J Invasive Cardiol.

[28] Olsson KM, Simon A, Strueber M, Hadem J, Wiesner O, Gottlieb J, Fuehner T, Fischer S, Warnecke G, Kuhn C, Haverich A, Welte T, Hoeper MM (2010). Extracorporeal membrane oxygenation in nonintubated patients as bridge to lung transplantation. Am J Transpl.

[29] Javidfar J, Brodie D, Takayama H, Mongero L, Zwischenberger J, Sonett J, Bacchetta M (2011). Safe transport of critically ill adult patients on extracorporeal membrane oxygenation support to a regional extracorporeal membrane oxygenation center. ASAIO J.

[30] Spina R, Forrest AP, Adams MR, Wilson MK, Ng MK, Vallely MP (2010). Veno-arterial extracorporeal membrane oxygenation for high-risk cardiac catheterisation procedures. Heart Lung Circ.

[31] Iliodromitis KE, Kahlert P, Plicht B, Hoffmann AC, Eggebrecht H, Erbel R, Konorza TF (2011). High-risk PCI in acute coronary syndromes with Impella LP 2.5 device support. Int J Cardiol.

[32] Haneya A, Philipp A, Puehler T, Ried M, Hilker M, Zink W, Hirt SW, Schmid C (2012). Ventricular assist device implantation in patients on percutaneous extracorporeal life support without switching to conventional cardiopulmonary bypass system. Eur J Cardio-thorac Surg.

[33] Tudorache I, Sommer W, Kühn C, Wiesner O, Hadem J, Fühner T, Ius F, Avsar M, Schwerk N, Böthig D, Gottlieb J, Welte T, Bara C, Haverich A, Hoeper MM, Warnecke G (2014) Lung transplantation for severe pulmonary hypertension-awake extracorporeal membrane oxygenation for postoperative left ventricular remodelling. Transplantation 99(2):451–458 10.1097/TP.0000000000000348 25119128

[34] Jaski BE, Ortiz B, Alla KR, Smith SC, Glaser D, Walsh C, Chillcott S, Stahovich M, Adamson R, Dembitsky W (2010). A 20-year experience with urgent percutaneous cardiopulmonary bypass for salvage of potential survivors of refractory cardiovascular collapse. J Thorac Cardiovasc Surg.

[35] Jung C, Janssen K, Kaluza M, Fuernau G, Poerner TC, Fritzenwanger M, Pfeifer R, Thiele H, Figulla HR (2015) Outcome predictors in cardiopulmonary resuscitation facilitated by extracorporeal membrane oxygenation. Clin Res Cardiol. doi:10.1007/s00392-015-0906-4 10.1007/s00392-015-0906-4 26303097

[36] Kagawa E, Dote K, Kato M, Sasaki S, Nakano Y, Kajikawa M, Higashi A, Itakura K, Sera A, Inoue I, Kawagoe T, Ishihara M, Shimatani Y, Kurisu S (2012). Should we emergently revascularize occluded coronaries for cardiac arrest? Rapid-response extracorporeal membrane oxygenation and intra-arrest percutaneous coronary intervention. Circulation.

[37] Napp LC, Brehm M, Kuhn C, Schafer A, Bauersachs J (2015). Heart against veno-arterial ECMO: competition visualized. Int J Cardiol.

[38] Cheng R, Hachamovitch R, Kittleson M, Patel J, Arabia F, Moriguchi J, Esmailian F, Azarbal B (2014). Complications of extracorporeal membrane oxygenation for treatment of cardiogenic shock and cardiac arrest: a meta-analysis of 1866 adult patients. Ann Thorac Surg.

[39] Bisdas T, Beutel G, Warnecke G, Hoeper MM, Kuehn C, Haverich A, Teebken OE (2011). Vascular complications in patients undergoing femoral cannulation for extracorporeal membrane oxygenation support. Ann Thorac Surg.

[40] Chung M, Shiloh AL, Carlese A (2014). Monitoring of the adult patient on venoarterial extracorporeal membrane oxygenation. Sci World J.

[41] Cheng A, Swartz MF, Massey HT (2013). Impella to unload the left ventricle during peripheral extracorporeal membrane oxygenation. ASAIO J.

[42] Javidfar J, Brodie D, Costa J, Miller J, Jurrado J, LaVelle M, Newmark A, Takayama H, Sonett JR, Bacchetta M (2012). Subclavian artery cannulation for venoarterial extracorporeal membrane oxygenation. ASAIO J.

[43] Choi JH, Kim SW, Kim YU, Kim SY, Kim KS, Joo SJ, Lee JS (2014). Application of veno-arterial-venous extracorporeal membrane oxygenation in differential hypoxia. Multidiscip Respir Med.

[44] Hou X, Yang X, Du Z, Xing J, Li H, Jiang C, Wang J, Xing Z, Li S, Li X, Yang F, Wang H, Zeng H (2015). Superior vena cava drainage improves upper body oxygenation during veno-arterial extracorporeal membrane oxygenation in sheep. Crit Care.

[45] Ford EG, Atkinson JB (1992). Augmented venous access in the problematic ECMO patient: a case report. J Pediatr Surg.

[46] Kustermann J, Gehrmann A, Kredel M, Wurmb T, Roewer N, Muellenbach RM (2013). Acute respiratory distress syndrome and septic cardiomyopathy: successful application of veno-venoarterial extracorporeal membrane oxygenation. Der Anaesth.

[47] Ius F, Sommer W, Tudorache I, Avsar M, Siemeni T, Salman J, Puntigam J, Optenhoefel J, Greer M, Welte T, Wiesner O, Haverich A, Hoeper M, Kuehn C, Warnecke G (2015). Veno-veno-arterial extracorporeal membrane oxygenation for respiratory failure with severe haemodynamic impairment: technique and early outcomes. Interact CardioVasc Thorac Surg.

[48] Moravec R, Neitzel T, Stiller M, Hofmann B, Metz D, Bucher M, Silber R, Bushnaq H, Raspe C (2014). First experiences with a combined usage of veno-arterial and veno-venous ECMO in therapy-refractory cardiogenic shock patients with cerebral hypoxemia. Perfusion.

[49] Chung JC, Tsai PR, Chou NK, Chi NH, Wang SS, Ko WJ (2010). Extracorporeal membrane oxygenation bridge to adult heart transplantation. Clin Transpl.

[50] Kim KI, Lee WY, Kim HS, Jeong JH, Ko HH (2014). Extracorporeal membrane oxygenation in near-drowning patients with cardiac or pulmonary failure. Scand J Trauma Resusc Emerg Med.

[51] Madershahian N, Wittwer T, Strauch J, Franke UF, Wippermann J, Kaluza M, Wahlers T (2007). Application of ECMO in multitrauma patients with ARDS as rescue therapy. J Card Surg.

[52] Biscotti M, Lee A, Basner RC, Agerstrand C, Abrams D, Brodie D, Bacchetta M (2014). Hybrid configurations via percutaneous access for extracorporeal membrane oxygenation: a single-center experience. ASAIO J.

[53] Stöhr F, Emmert MY, Lachat ML, Stocker R, Maggiorini M, Falk V, Wilhelm MJ (2011). Extracorporeal membrane oxygenation for acute respiratory distress syndrome: is the configuration mode an important predictor for the outcome?. Interact CardioVasc Thorac Surg.

